# Supplementary material for: Survival outcomes of surgery for retroperitoneal sarcomas: A systematic review and meta-analysis
Source: PLoS One. 2022 Jul 28;17(7):e0272044. doi: 10.1371/journal.pone.0272044 (PMC9333279; doi:10.1371/journal.pone.0272044)
Supplement: S1 File — (DOC) [file pone.0272044.s001.doc]

**Supporting Information – contains all the supporting tables and figures**

**S1 Text. Search strategy**

**S1 Table. Quality analyses of the trials included in the systematic review and meta-analysis**

**S2 Table. PRISMA checklist**

**S1 Fig. Pooled complication rate of extended resection versus tumour resection alone**

**S2 Fig. Pooled mortality rate of extended resection versus tumour resection alone**

**S3 Fig. Pooled disease-free survival of extended resection versus tumour resection alone**

**S4 Fig. Subgroup analysis disease-free survival of extended resection versus tumour resection alone (primary RPS)**

**S5 Fig. Subgroup analysis disease-free survival of extended resection versus tumour resection alone (recurrent RPS)**

**S6 Fig. Pooled over-all survival of extended resection versus tumour resection alone**

**S7 Fig. Subgroup analysis over-all survival of extended resection versus tumour resection alone (primary RPS)**

**S8 Fig. Subgroup analysis over-all survival of extended resection versus tumour resection alone (recurrent RPS)**

**S9 Fig. Pooled over-all survival of R0 vs R1**

**S10 Fig. Subgroup analysis of over-all survival of R0 vs R1 (primary RPS)**

**S11 Fig. Pooled over-all survival of R1 vs R2**

**S12 Fig. Subgroup analysis of over-all survival of R1 vs R2 (primary RPS)**

**S13 Fig. Pooled over-all survival of R2 vs no surgery**

**S14 Fig. Subgroup analysis of over-all survival of R2 vs no surgery (primary RPS)**

**Text S1: Search strategy**

**Medline search strategy**

1. exp retroperitoneal sarcoma/
2. exp RPS /
3. exp retroperitoneal tumour /
4. exp retroperitoneal neoplasm/
5. (rps* or (retroperit$ adj8 sarcoma$ adj8).mp. [mp=title, original title, abstract, name of substance word, subject heading word, unique identifier]
6. 1 or 2 or 3 or 4 or 5
7. surgery/
8. surg$.tw.
9. resect$.tw.
10. operati$.tw.
11. intervent$.tw.
12. 7 or 8 or 9 or 10 or 11 or 12
13. 6 and 12
14. limit 13 to humans

**Table S1: Quality analyses of the trials included in the systematic review and meta-analysis**

| Studies | Selection | Comparability | Outcome | Total |
| --- | --- | --- | --- | --- |
| Abdelfatah 2016^17^ | 4 | 0 | 3 | 7 |
| Bagaria 2018^18^ | 4 | 0 | 3 | 7 |
| Bengmark 1990^19^ | 4 | 2 | 3 | 9 |
| Bonvalot 2008^20^ | 4 | 0 | 3 | 7 |
| Bremjit 2014^21^ | 4 | 0 | 3 | 7 |
| Chiappa 2006^22^ | 4 | 0 | 3 | 7 |
| Chiappa 2018^23^ | 4 | 1 | 3 | 8 |
| Doepker 2016^24^ | 4 | 0 | 3 | 7 |
| Erzen 2005^25^ | 4 | 0 | 3 | 7 |
| Fujimoto 2018^26^ | 4 | 0 | 3 | 7 |
| Garcı´a-Aceituno 2010^27^ | 4 | 0 | 3 | 7 |
| Gilbeau 2002^28^ | 4 | 0 | 3 | 7 |
| Grobmyer 2010^29^ | 4 | 0 | 3 | 7 |
| Gronchi 2014^30^ | 4 | 0 | 3 | 7 |
| Ikoma 2017^31^ | 4 | 0 | 3 | 7 |
| Ikoma 2018^10^ | 4 | 2 | 3 | 9 |
| Ishii 2020^32^ | 4 | 0 | 3 | 7 |
| Jaques 1989^33^ | 4 | 1 | 3 | 8 |
| Karakousis 1985^34^ | 4 | 0 | 3 | 7 |
| Lehnert 2009^35^ | 4 | 0 | 3 | 7 |
| Lewis 1998^36^ | 4 | 0 | 3 | 7 |
| Lochan 2011^37^ | 4 | 0 | 3 | 7 |
| Lu 2013^38^ | 4 | 1 | 3 | 8 |
| MacNeill 2017^39^ | 4 | 0 | 3 | 7 |
| Martin 2020^40^ | 4 | 0 | 3 | 7 |
| McGrath 1984^41^ | 4 | 0 | 3 | 7 |
| Milone 2011^42^ | 4 | 1 | 3 | 8 |
| Miura 2015^43^ | 4 | 0 | 3 | 7 |
| Morizawa 2006^44^ | 4 | 1 | 3 | 8 |
| Mussi 2011^45^ | 4 | 0 | 3 | 7 |
| Nathenson 2018^46^ | 4 | 0 | 3 | 7 |
| Pinson 1989^47^ | 4 | 0 | 3 | 7 |
| Rhu 2019^48^ | 4 | 0 | 3 | 7 |
| Roeder 2017^49^ | 4 | 0 | 3 | 7 |
| Rossi 2013^50^ | 4 | 0 | 3 | 7 |
| Santos 2010^51^ | 4 | 2 | 3 | 9 |
| Shibata 2001^12^ | 4 | 0 | 3 | 7 |
| Shiloni 1993^52^ | 4 | 0 | 3 | 7 |
| Singer 2003^53^ | 4 | 0 | 3 | 7 |
| Tan 2016^54^ | 4 | 0 | 3 | 7 |
| Thalji 2020^55^ | 4 | 0 | 3 | 7 |
| Tropea 2020^56^ | 4 | 0 | 3 | 7 |
| Tseng 2010^57^ | 4 | 0 | 3 | 7 |
| van Houdt 2020^58^ | 4 | 1 | 3 | 8 |
| Villano 2020^59^ | 4 | 2 | 3 | 9 |
| Yang 2014^60^ | 4 | 0 | 3 | 7 |
| Zhao 2015^61^ | 4 | 0 | 3 | 7 |

**Table S2: PRISMA checklist**

| **Section/topic** | **#** | **Checklist item** | **Reported on page #** |
| --- | --- | --- | --- |
| **TITLE** | | |  |
| Title | 1 | Identify the report as a systematic review, meta-analysis, or both. | #1 |
| **ABSTRACT** | | |  |
| Structured summary | 2 | Provide a structured summary including, as applicable: background; objectives; data sources; study eligibility criteria, participants, and interventions; study appraisal and synthesis methods; results; limitations; conclusions and implications of key findings; systematic review registration number. | #2 |
| **INTRODUCTION** | | |  |
| Rationale | 3 | Describe the rationale for the review in the context of what is already known. | #3 |
| Objectives | 4 | Provide an explicit statement of questions being addressed with reference to participants, interventions, comparisons, outcomes, and study design (PICOS). | #3 |
| **METHODS** | | |  |
| Protocol and registration | 5 | Indicate if a review protocol exists, if and where it can be accessed (e.g., Web address), and, if available, provide registration information including registration number. | NS |
| Eligibility criteria | 6 | Specify study characteristics (e.g., PICOS, length of follow-up) and report characteristics (e.g., years considered, language, publication status) used as criteria for eligibility, giving rationale. | #3,4 |
| Information sources | 7 | Describe all information sources (e.g., databases with dates of coverage, contact with study authors to identify additional studies) in the search and date last searched. | #3 |
| Search | 8 | Present full electronic search strategy for at least one database, including any limits used, such that it could be repeated. | #3 |
| Study selection | 9 | State the process for selecting studies (i.e., screening, eligibility, included in systematic review, and, if applicable, included in the meta-analysis). | #3,4 |
| Data collection process | 10 | Describe method of data extraction from reports (e.g., piloted forms, independently, in duplicate) and any processes for obtaining and confirming data from investigators. | #4 |
| Data items | 11 | List and define all variables for which data were sought (e.g., PICOS, funding sources) and any assumptions and simplifications made. | #4 |
| Risk of bias in individual studies | 12 | Describe methods used for assessing risk of bias of individual studies (including specification of whether this was done at the study or outcome level), and how this information is to be used in any data synthesis. | #4 |
| Summary measures | 13 | State the principal summary measures (e.g., risk ratio, difference in means). | #4 |
| Synthesis of results | 14 | Describe the methods of handling data and combining results of studies, if done, including measures of consistency (e.g., I^2^) for each meta-analysis. | #4 |

Page 1 of 2

| **Section/topic** | **#** | **Checklist item** | **Reported on page #** |
| --- | --- | --- | --- |
| Risk of bias across studies | 15 | Specify any assessment of risk of bias that may affect the cumulative evidence (e.g., publication bias, selective reporting within studies). | #4 |
| Additional analyses | 16 | Describe methods of additional analyses (e.g., sensitivity or subgroup analyses, meta-regression), if done, indicating which were pre-specified. | #4 |
| **RESULTS** | | |  |
| Study selection | 17 | Give numbers of studies screened, assessed for eligibility, and included in the review, with reasons for exclusions at each stage, ideally with a flow diagram. | #4 |
| Study characteristics | 18 | For each study, present characteristics for which data were extracted (e.g., study size, PICOS, follow-up period) and provide the citations. | #4,5,Appendix |
| Risk of bias within studies | 19 | Present data on risk of bias of each study and, if available, any outcome level assessment (see item 12). | #4 |
| Results of individual studies | 20 | For all outcomes considered (benefits or harms), present, for each study: (a) simple summary data for each intervention group (b) effect estimates and confidence intervals, ideally with a forest plot. | #4,5 |
| Synthesis of results | 21 | Present results of each meta-analysis done, including confidence intervals and measures of consistency. | #5,6,Appendix |
| Risk of bias across studies | 22 | Present results of any assessment of risk of bias across studies (see Item 15). | #5 |
| Additional analysis | 23 | Give results of additional analyses, if done (e.g., sensitivity or subgroup analyses, meta-regression [see Item 16]). | #5,6 |
| **DISCUSSION** | | |  |
| Summary of evidence | 24 | Summarize the main findings including the strength of evidence for each main outcome; consider their relevance to key groups (e.g., healthcare providers, users, and policy makers). | #6 |
| Limitations | 25 | Discuss limitations at study and outcome level (e.g., risk of bias), and at review-level (e.g., incomplete retrieval of identified research, reporting bias). | #8 |
| Conclusions | 26 | Provide a general interpretation of the results in the context of other evidence, and implications for future research. | #8 |
| **FUNDING** | | |  |
| Funding | 27 | Describe sources of funding for the systematic review and other support (e.g., supply of data); role of funders for the systematic review. | #8 |

*From:*  Moher D, Liberati A, Tetzlaff J, Altman DG, The PRISMA Group (2009). Preferred Reporting Items for Systematic Reviews and Meta-Analyses: The PRISMA Statement. PLoS Med 6(6): e1000097. doi:10.1371/journal.pmed1000097

For more information, visit: **www.prisma-statement.org**. Page 2 of 2

**Figure S1: Pooled complication rate of extended resection versus tumour resection alone**

**
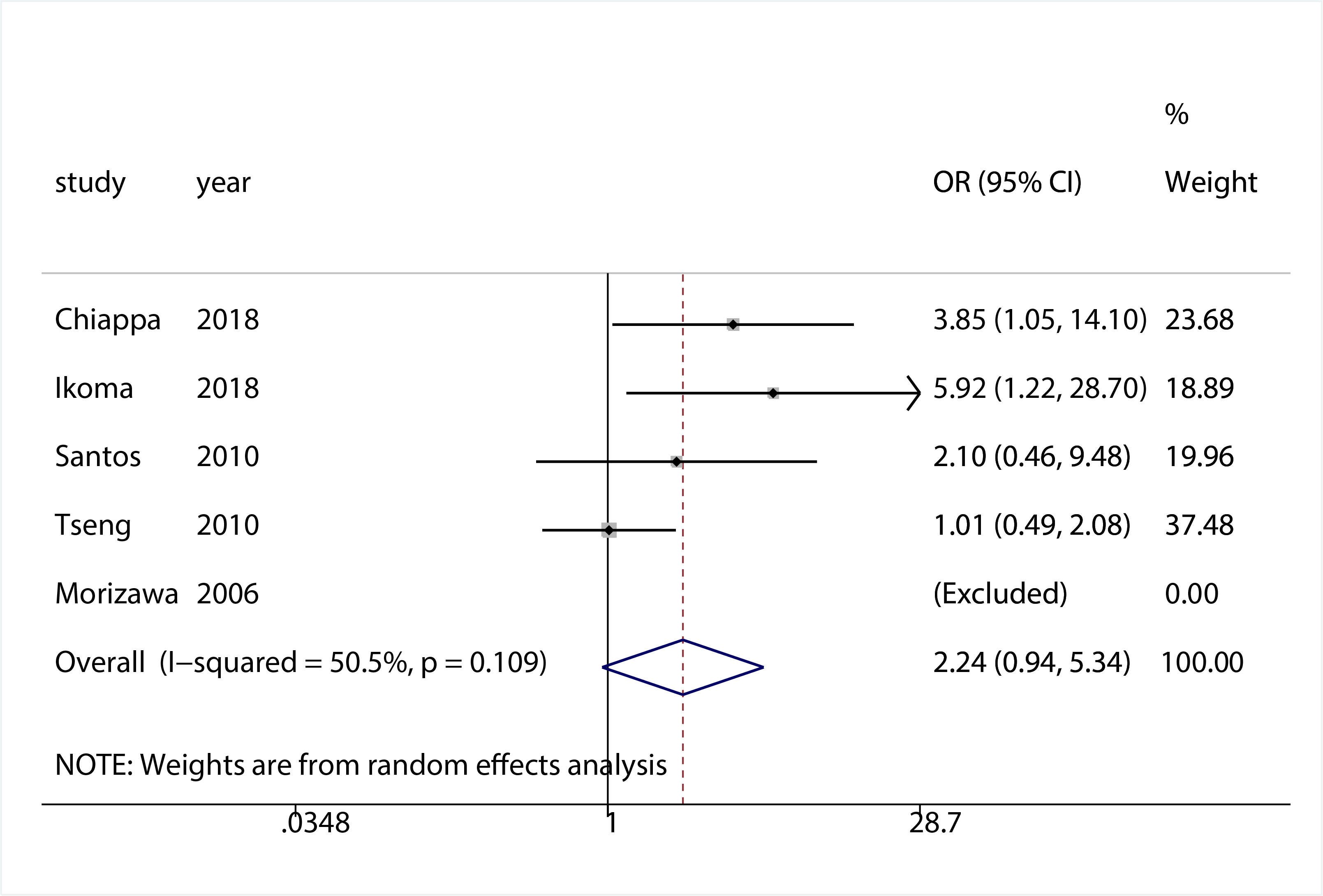
**

**Figure S2: Pooled mortality rate of extended resection versus tumour resection alone**

**
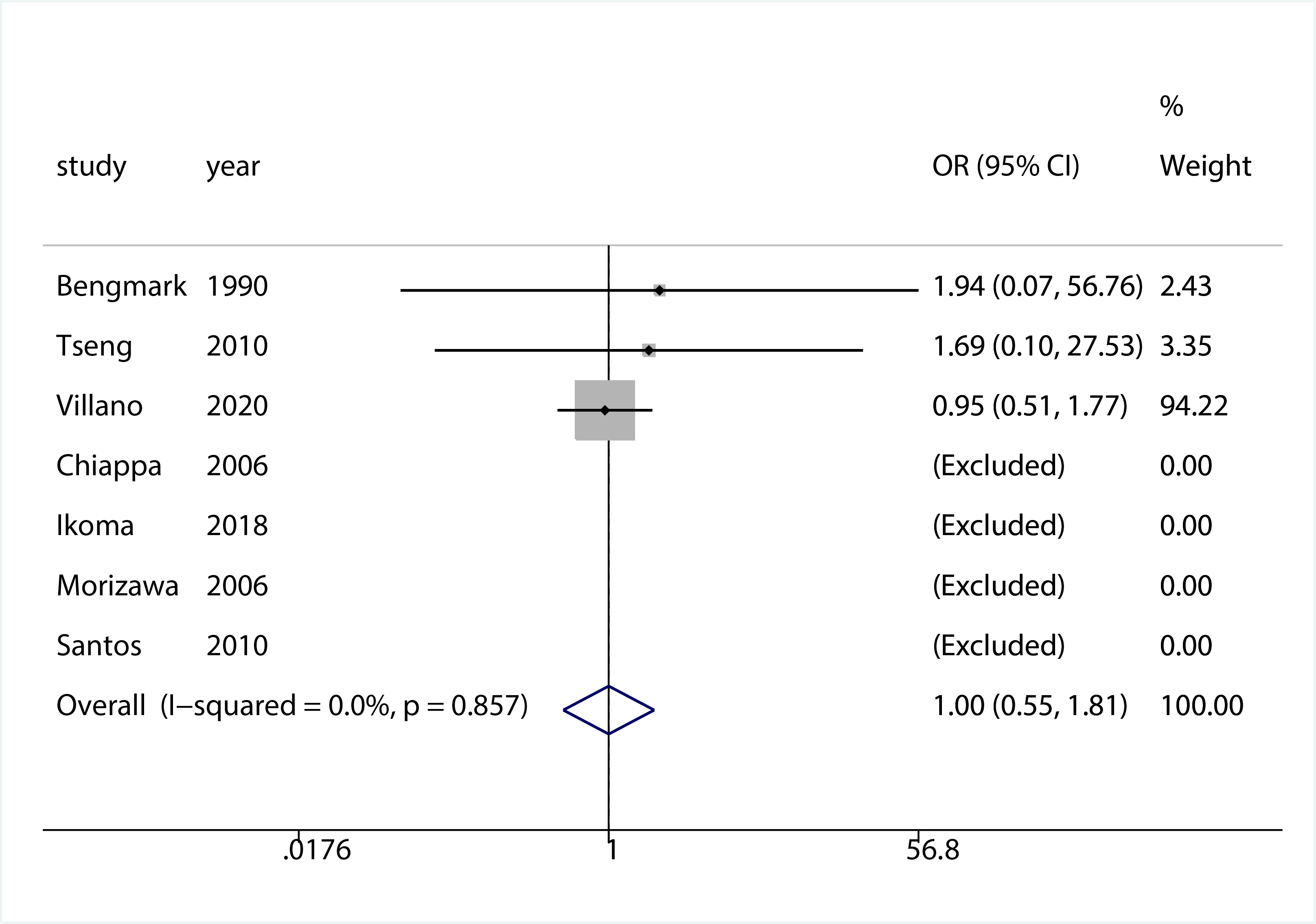
**

**Figure S3: Pooled disease-free survival of extended resection versus tumour resection alone**

**
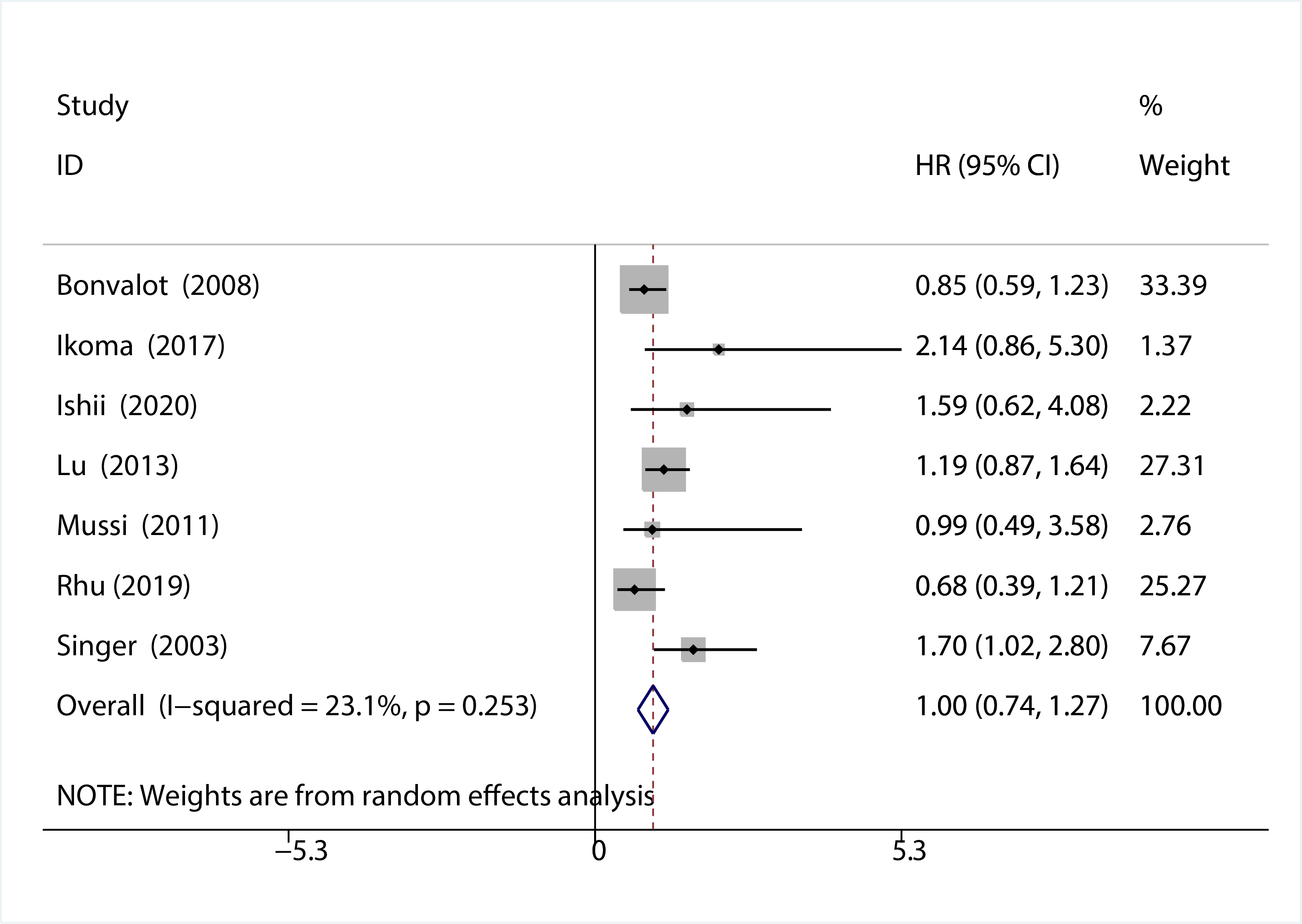
**

**Figure S4: Subgroup analysis disease-free survival of extended resection versus tumour resection alone (primary RPS)**

**
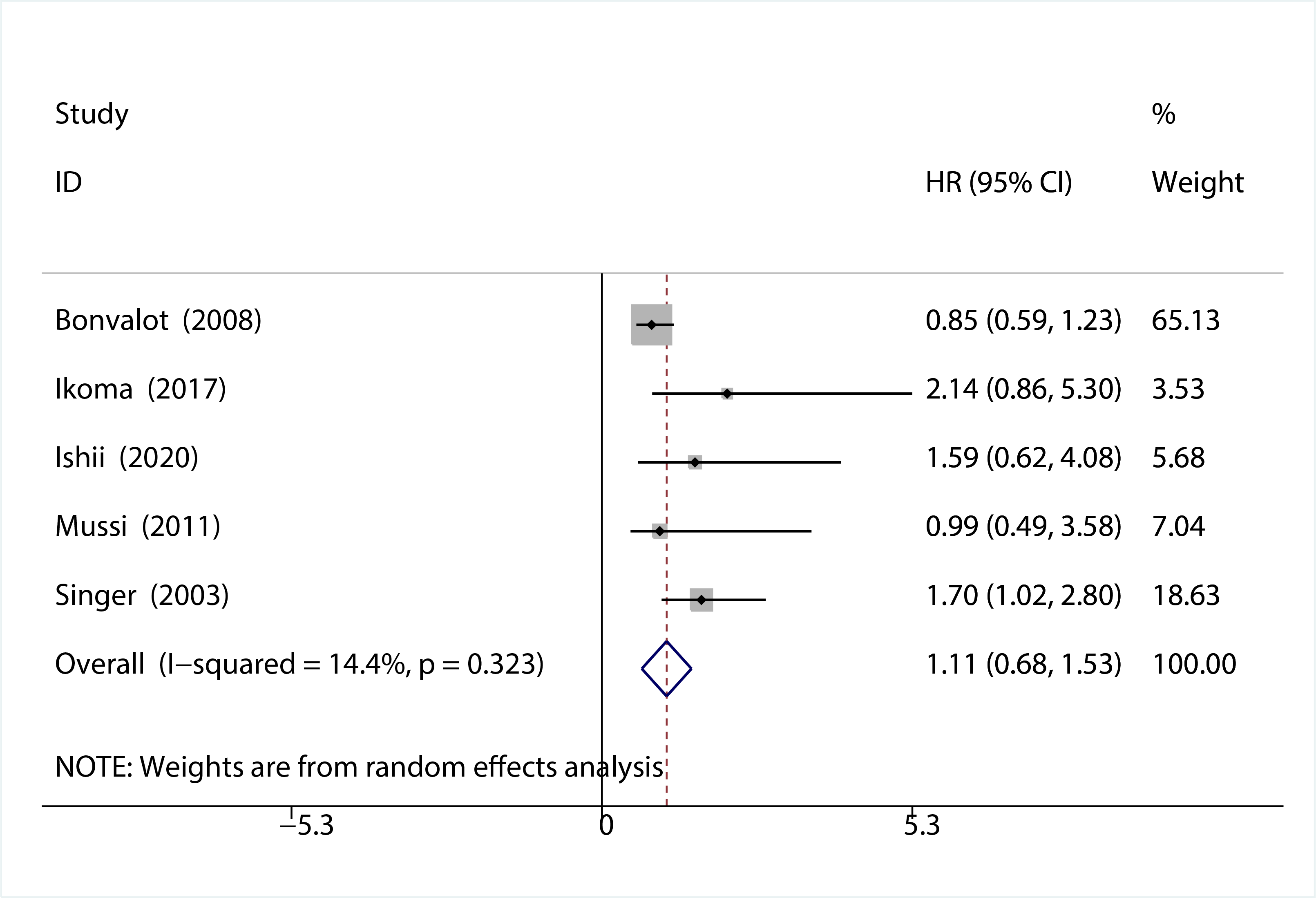
**

**Figure S5: Subgroup analysis disease-free survival of extended resection versus tumour resection alone (recurrent RPS)**

**
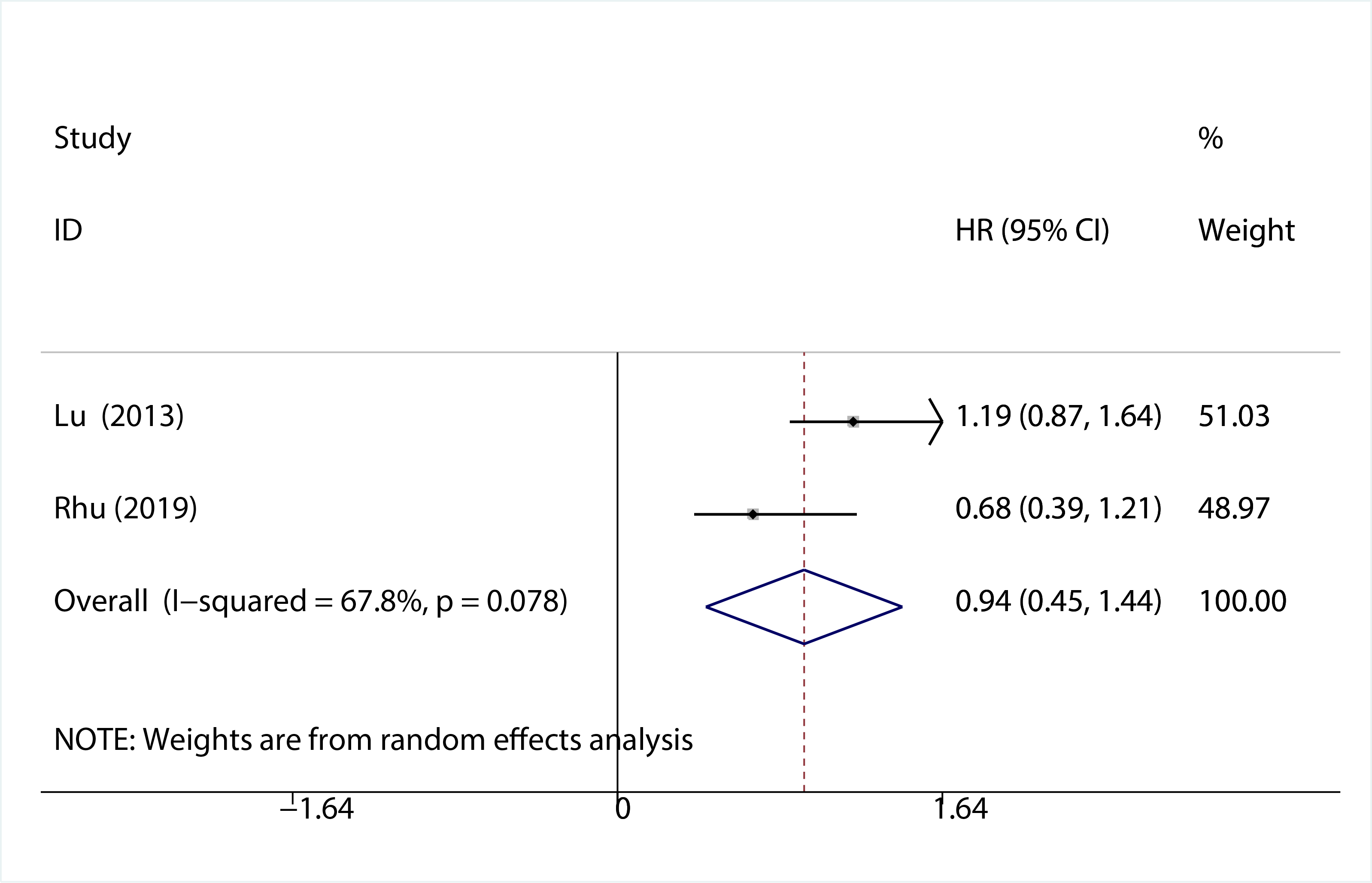
**

**Figure S6: Pooled over-all survival of extended resection versus tumour resection alone**

**
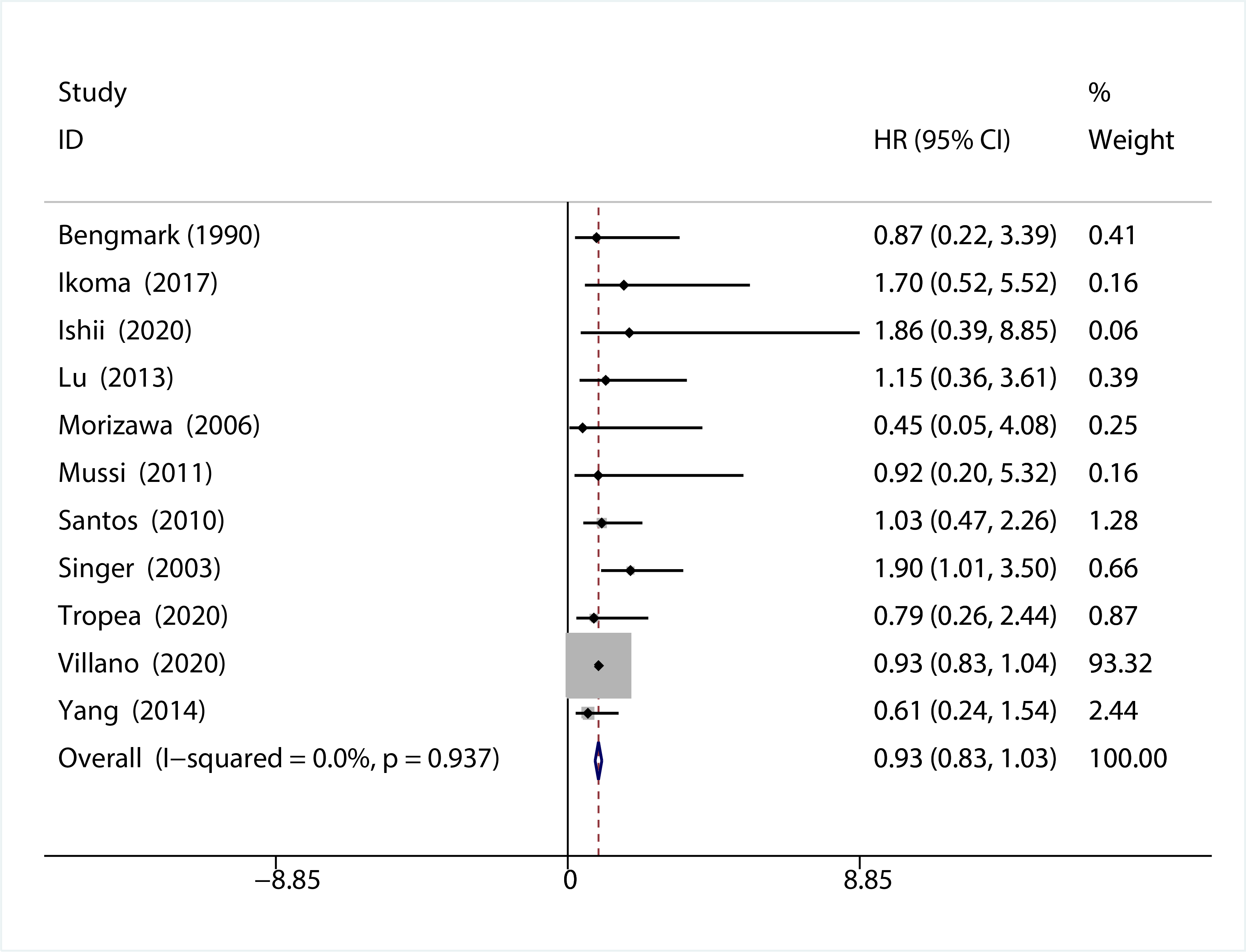
**

**Figure S7: Subgroup analysis over-all survival of extended resection versus tumour resection alone (primary RPS)**

**
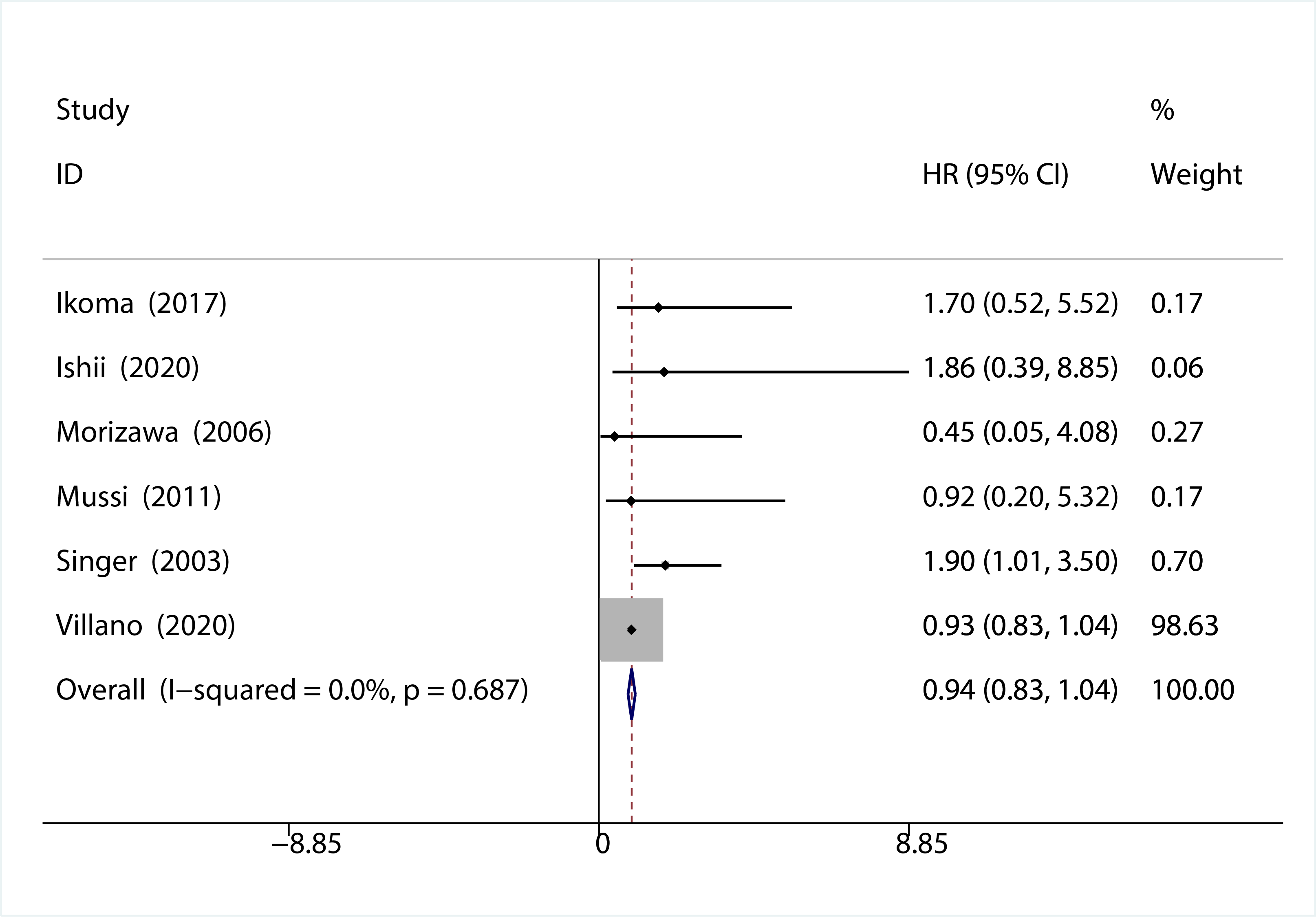
**

**Figure S8: Subgroup analysis over-all survival of extended resection versus tumour resection alone (recurrent RPS)**

**
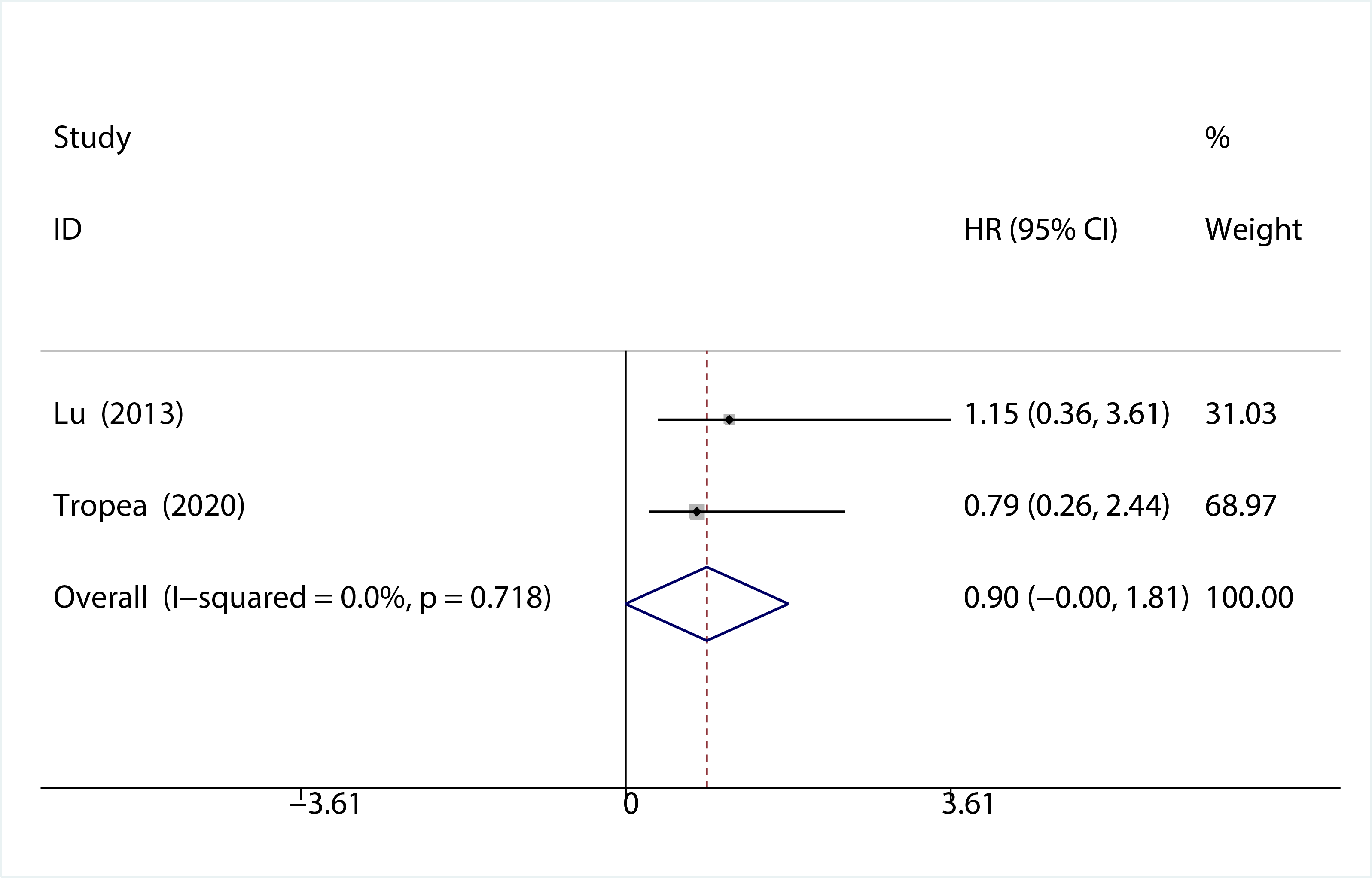
**

**Figure S9: Pooled over-all survival of R0 vs R1**

**
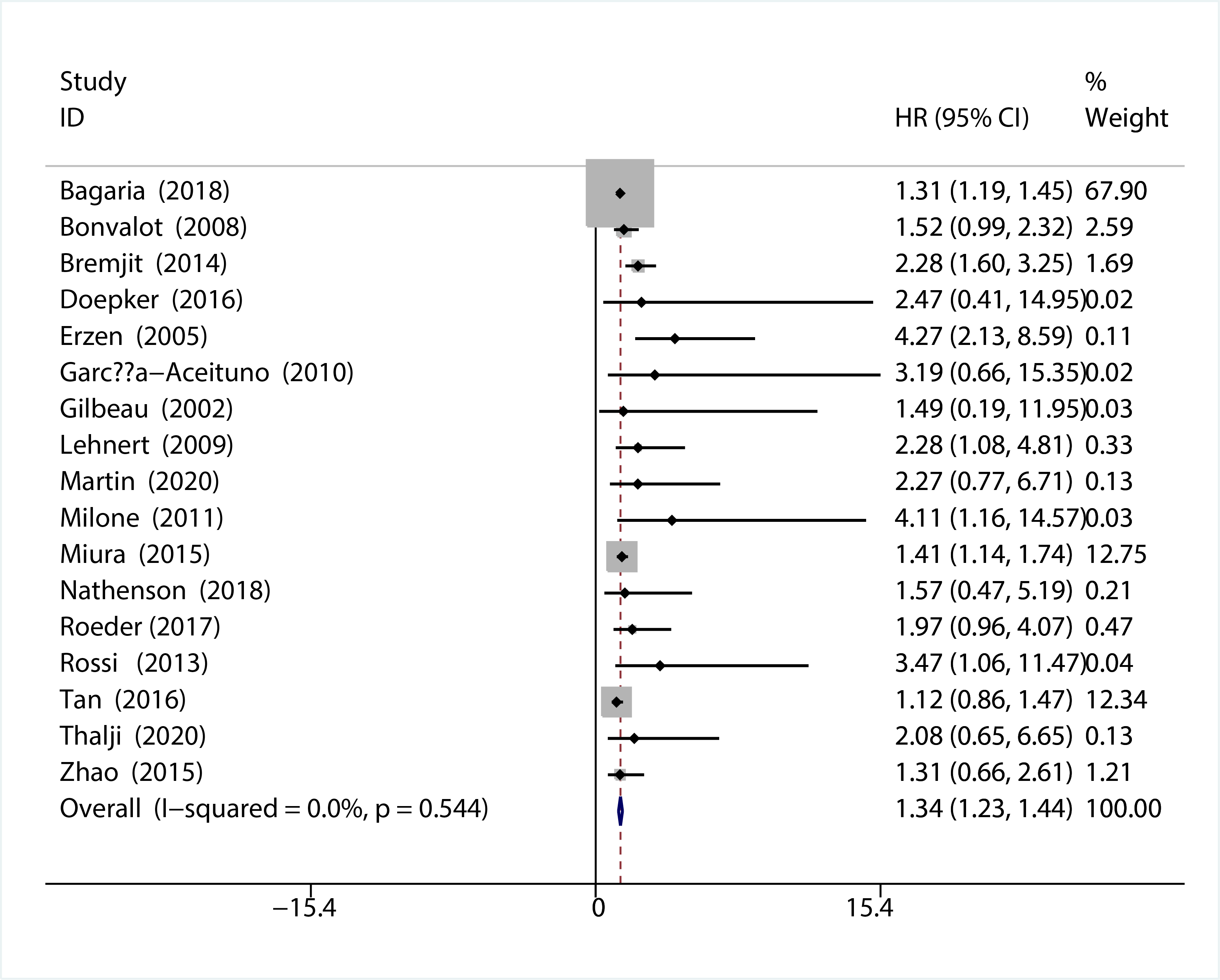
**

**Figure S10: Subgroup analysis of over-all survival of R0 vs R1 (primary RPS)**

**
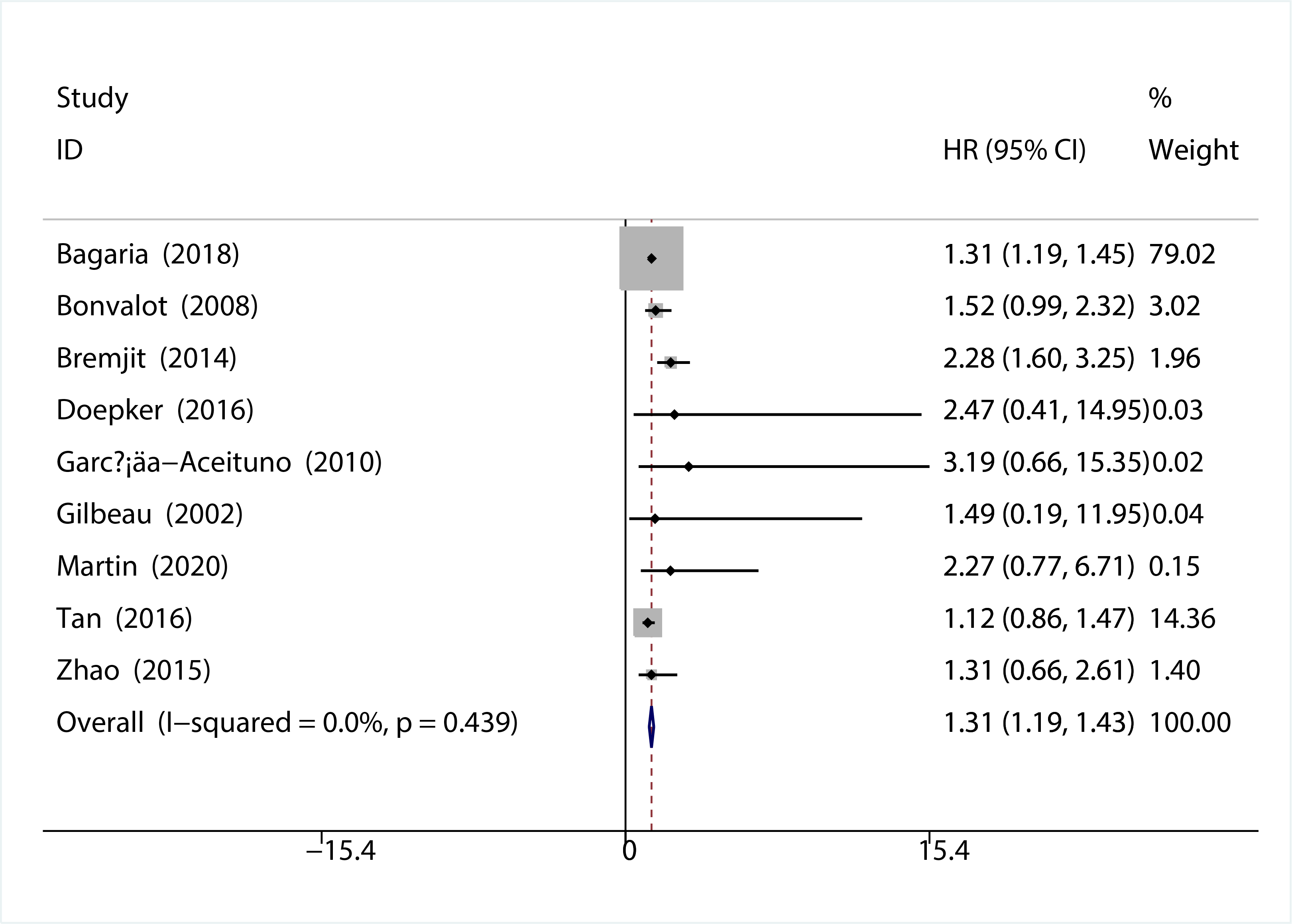
**

**Figure S11: Pooled over-all survival of R1 vs R2**

**
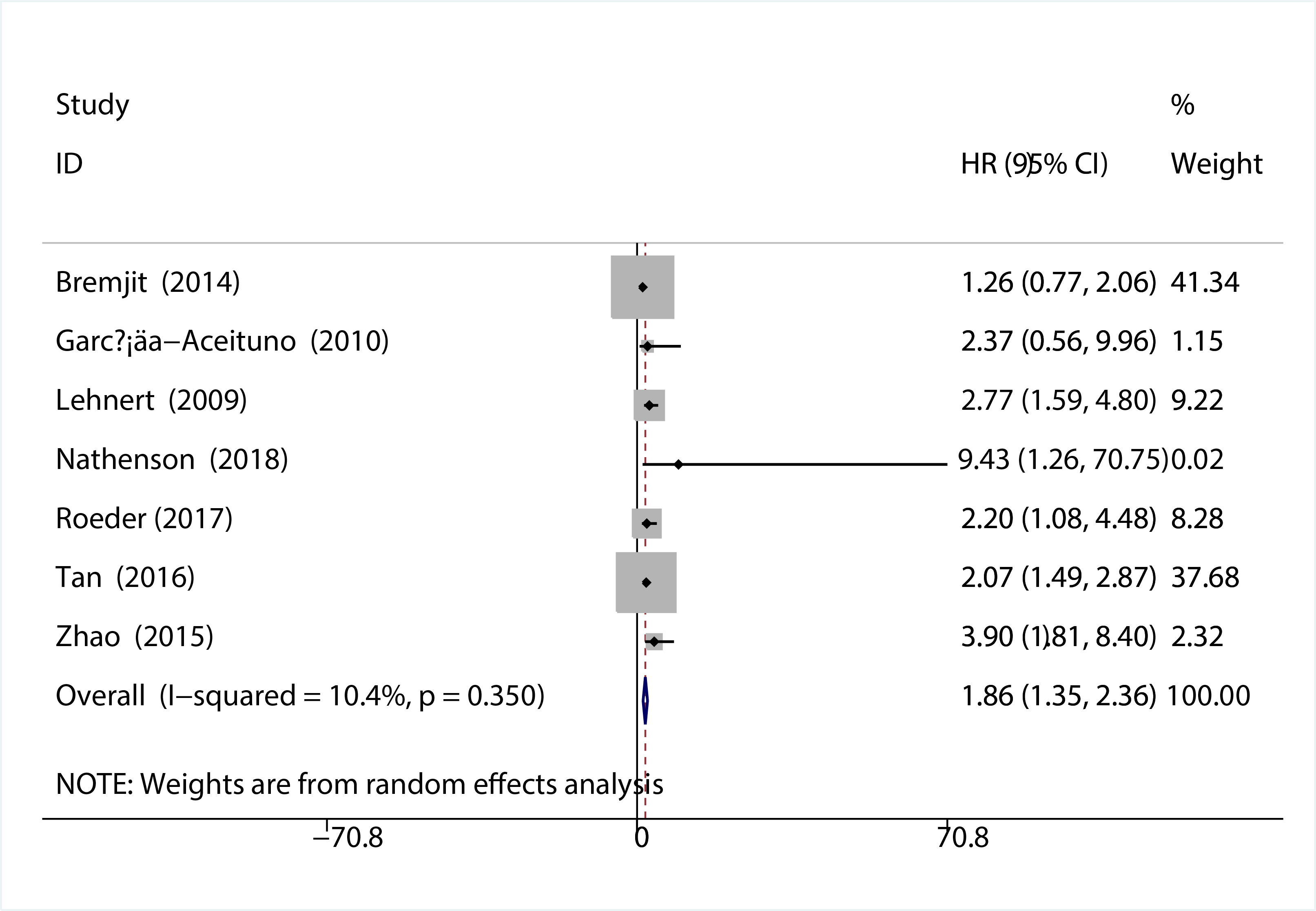
**

**Figure S12: Subgroup analysis of over-all survival of R1 vs R2 (primary RPS)**

**
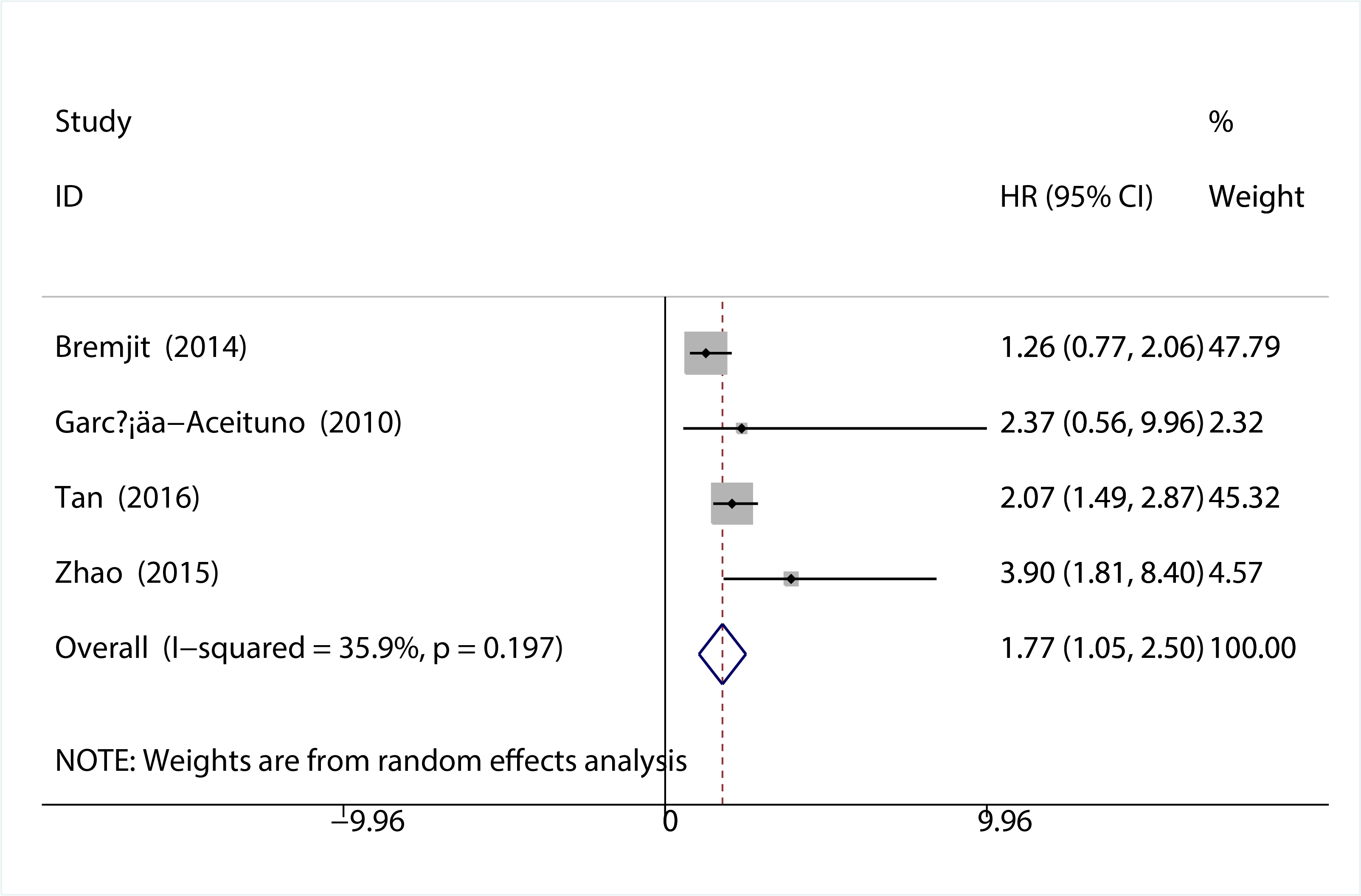
**

**Figure S13: Pooled over-all survival of R2 vs no surgery**

**
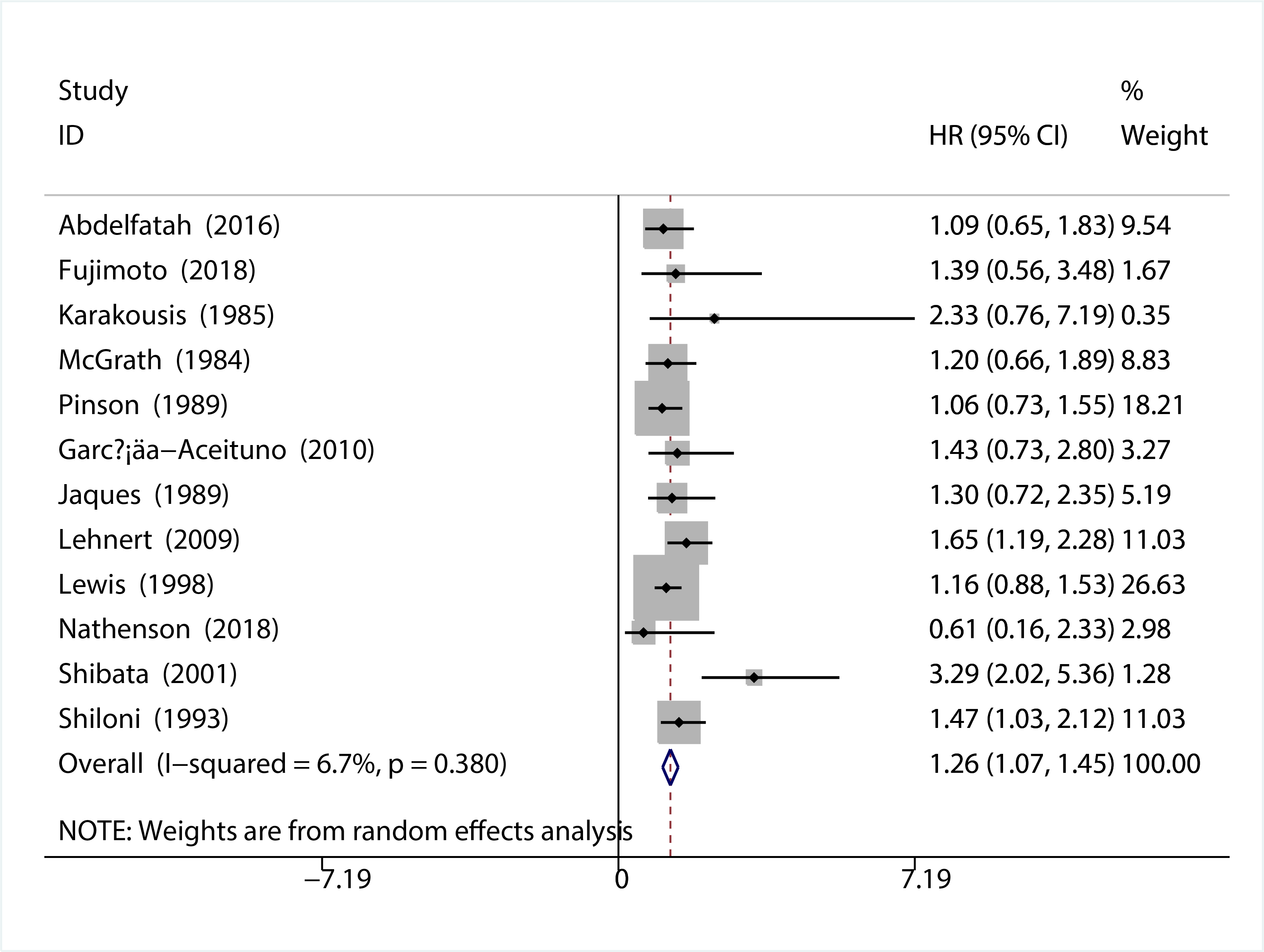
**

**Figure S14: Subgroup analysis of over-all survival of R2 vs no surgery (primary RPS)**

**
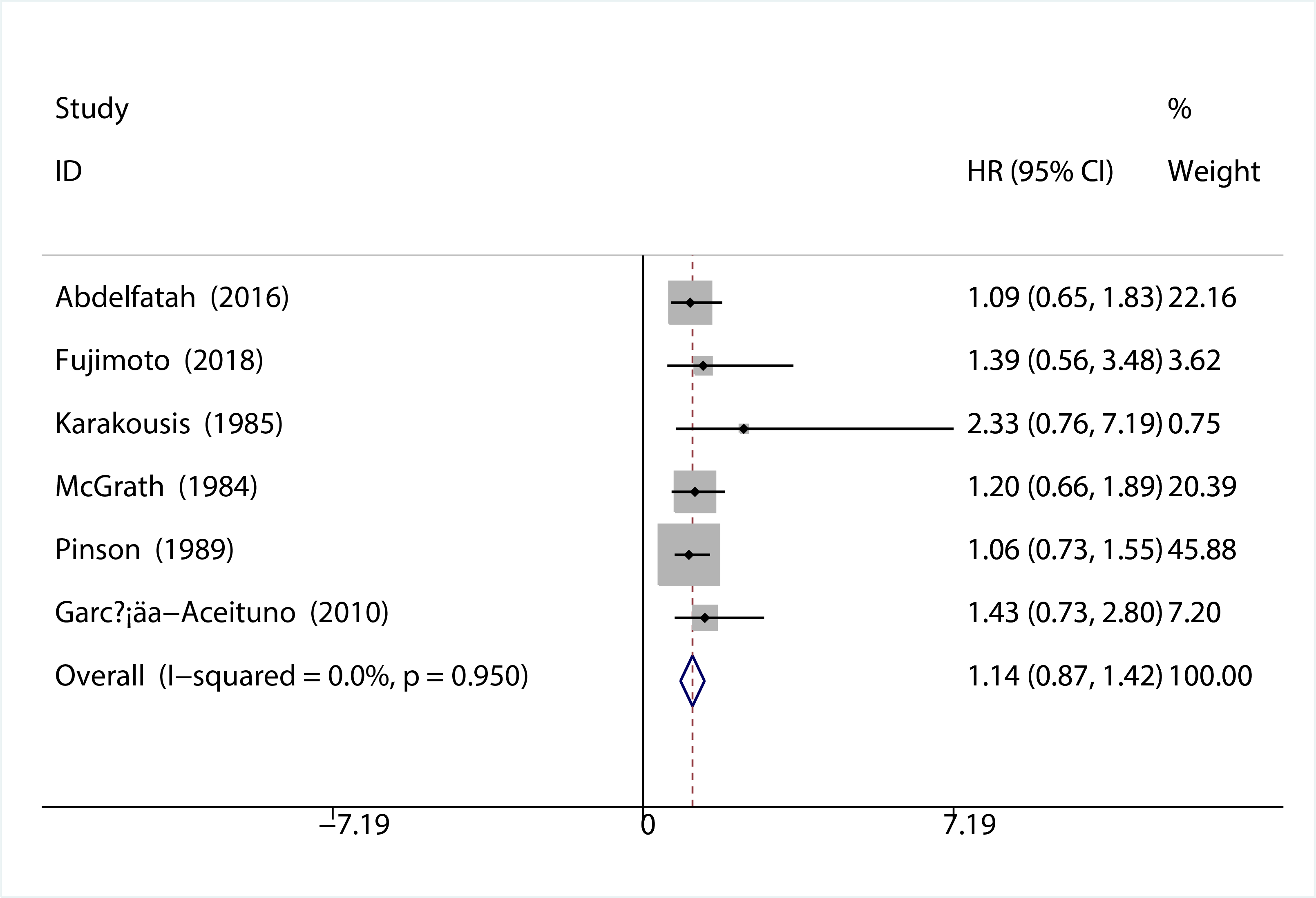
**
