# Supplementary material for: Survival outcomes of surgery for retroperitoneal sarcomas: A systematic review and meta-analysis
Source: PLoS One. 2022 Jul 28;17(7):e0272044. doi: 10.1371/journal.pone.0272044 (PMC9333279; doi:10.1371/journal.pone.0272044)
Supplement: Attachment — Submitted filename: plos review-retroperitoneal sarcoma.doc [file pone.0272044.s002.doc]

Review

Survival Outcomes of Surgery for Retroperitoneal Sarcomas: A Systematic Review and Meta-analysis

Abstract – lacking some relevant sections. Study objectives were not stated. The abstract ought to necessarily state the inclusion/exclusion criteria for the articles used in the study. This was absent.

Introduction – a good background was laid for the study. The objectives are clearly stated and defined.

Methods – a good description of the inclusion/exclusion was provided. Search strategies were comprehensively explained. Attempts were made to determine the heterogeneity and the methodological qualities of the studies included.

Results – In the write up, the authors stated they included 47 articles in the meta-analysis. However, from the categorization, one will realize that there were 48 in all and not 47. 17 compared extended resection to tumor alone, 26 reported on surgical margins and 5 reported on long term outcomes.

Extended Resection vrs Tumor resection alone

The authors compared complications between extended resection and tumor resection alone and found no difference. It will be useful to know the complications common to both groups.

Surgical resection margins

The section highlighted the importance of complete tumor resection. Patients with negative surgical margins had a better overall survival compared to those with positive surgeons. The distinction was even clearer when whose with R1 resection were compared to R2 resections. R1 resected tumors had a better overall survival than R2 tumors.

Even more, recurrent tumors managed by surgical intervention had a better long term survival compared to those managed conservatively.

Discussion

The discussion highlighted the importance of surgical resection, even in advance and recurrent tumors. It was demonstrated that overall survival even in R2 resected tumors were higher than those managed conservatively.

With regards to extended resection and the tumor resection alone groups, it would have been interesting to determine the rate of negative surgical margins in both groups since they seem to have similar morbidity, mortality and disease free survival rates. Is the surgical margin a determinant of the similar mortality and disease free rate? The authors could perform a sub-group analysis looking at this factor.

Conclusion

The conclusion was derived from the study findings and therefore appropriate

Other comments

A sub-group analysis looking at the role of radiotherapy and chemotherapy, and its influence on overall survival and disease free survival could have enriched the study findings. They could have also explored the role of neo-adjuvant vrs adjuvant therapy in the management of these cancers.

Recommendation

I recommend the publication of this manuscript subject to modifications based on the comments made above. The authors may choose not to respond to comments made under ‘other comments’.
